# Black Scoter habitat use along the southeastern coast of the United States

**DOI:** 10.1002/ece3.7746

**Published:** 2021-07-27

**Authors:** Hannah M. Plumpton, Emily D. Silverman, Beth E. Ross

**Affiliations:** ^1^ Department of Forestry and Environmental Conservation Clemson University Clemson SC USA; ^2^ Division of Migratory Bird Management U.S. Fish and Wildlife Service Laurel MD USA; ^3^ South Carolina Cooperative Fish and Wildlife Research Unit U.S. Geological Survey Clemson University Clemson SC USA

**Keywords:** Black Scoter, habitat use, *Melanitta americana*, oceanographic, weather

## Abstract

While the Atlantic Coast of the United States and Canada is a major wintering area for sea ducks, knowledge about their wintering habitat use is relatively limited. Black Scoters have a broad wintering distribution and are the only open water species of sea duck that is abundant along the southeastern coast of the United States. Our study identified variables that affected Black Scoter (*Melanitta americana*) distribution and abundance in the Atlantic Ocean along the southeastern coast of the United States. We used aerial survey data from 2009 to 2012 provided by the United States Fish and Wildlife Service to identify variables that influenced Black Scoter distribution. We used indicator variable selection to evaluate relationships between Black Scoter habitat use and a variety of broad‐ and fine‐scale oceanographic and weather variables. Average time between waves, ocean floor slope, and the interaction of bathymetry and distance to shore had the strongest association with southeastern Black Scoter distribution.

## Graphical abstract

Our study identified variables that had the most influence on Black Scoter distribution in the Atlantic Ocean along the southeastern coast of the United States. We used variable selection to evaluate relationships between Black Scoter habitat use and a variety of broad‐ and fine‐scale oceanographic and weather variables.

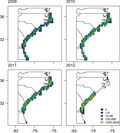

## 1 INTRODUCTION

The quality and quantity of habitats and resources during the nonbreeding season may be important limiting factors for waterfowl (Fretwell, 1972; Lack, 1966; Sedinger & Alisauskas, 2014) and affect population dynamics (Alisauskas & Ankney, 1992; Martin & Wiebe, 2004; Scott, 1998). Poor winter habitat conditions have been associated with large mortality events (Camphuysen et al., 2002), decreased reproductive success (Guillemain et al., 2008; Nichols et al., 1983; Oosterhuis & Dijk, 2002), and decreased population growth rate (Petersen & Douglas, 2004; Saino et al., 2004; Sorensen et al., 2009). Despite the importance of high‐quality wintering habitat, there is limited information on winter habitat use by sea ducks, particularly along the Atlantic Coast of the United States (Boyd et al., 2015; Jodice et al., 2013; Kaplan, 2011; Sea Duck Joint Venture, 2015).

Sea duck distributions within the wintering range may be based on a variety of factors including local environmental conditions (Beuth et al., 2017; Loring et al., 2014), food availability (Guillemette et al., 1993; Lewis et al., 2008), predation risk, site fidelity (Greenwood, 1980), and human activity (Madsen & Fox, 1995). Wintering distributions are also likely influenced by fine (<2 km) and broad‐scale (>2 km) oceanographic and weather conditions (Silverman et al., 2013a; Zipkin et al., 2010). The North Atlantic Oscillation (NAO; Zipkin et al., 2010), distance to shore (Goudie & Ankney, 1988; Loring et al., 2014), bathymetry (Plumpton et al., 2020), ocean floor slope (Silverman et al., 2013a; Zipkin et al., 2010), wind speed, and time between waves (Palm et al., 2013) all affect sea duck wintering distributions. While these oceanographic and environmental variables affect sea duck distributions along the northern Atlantic Coast of the United States, as these conditions change in more southernly habitats, sea ducks wintering further south may shift their habitat use (Plumpton et al., 2020).

While most sea duck species winter north of the South Atlantic Bight (Lamb et al., 2020), Black Scoters range from the coast of Canada down to Florida. As their range shifts from northern to southern habitats, the response of Black Scoters to environmental conditions at these southern latitudes differs from their response to conditions at more northern locations (Plumpton et al., 2020). Additionally, the species exhibits high interannual variation in wintering habitats, making monitoring their wintering populations challenging (Silverman et al., 2013a). A better understanding of the range of conditions that Black Scoters use during the wintering season in the southern portion of their range may provide important insights into management of the species and highlight potential areas of conservation concern, such as development of offshore wind farms.

In an effort to quantify the abundance and wintering distribution of sea duck populations along the Atlantic Coast, the U.S. Fish and Wildlife Service (USFWS) initiated the Atlantic Coast Wintering Sea Duck survey in 2008 and conducted aerial surveys from 2009 to 2012 along the Atlantic Coast of the United States (Silverman et al., 2013a). These surveys focused primarily on five species of concern due to current population declines, potential harvest implications, or habitat limitations (Sea Duck Joint Venture, 2015). The species surveyed were the Common Eider (*Somateria mollissima*), Long‐tailed Duck (*Clangula hyemalis*), Surf Scoter (*Melanitta perspicillata*), White‐winged Scoter (*Melanitta fusca*), and Black Scoter (*Melanitta americana*).

To better understand drivers of wintering distribution and abundance of Black Scoters along the southern Atlantic Coast of the United States, we used aerial survey data and a variety of broad‐ and fine‐scale oceanographic and climatic covariates. Our data incorporate a broad range of latitudes, providing an assessment of Black Scoter distribution in relation to variables in more southernly locations. We built upon Silverman et al. (2013a) to examine both oceanographic and climatic variables at a local and regional scale using all available survey data (including additional intensive surveying at more southern latitudes in 2012). We predicted that some habitats might be similar in more northern areas of the Atlantic (e.g., distance to shore), and unique habitats along the Southern Atlantic Coast would result in habitat differences (e.g., bathymetry and ocean floor slope).

## 2 METHODS

### 2.1 Survey design

The U.S. Fish and Wildlife Service conducted aerial offshore winter surveys from 2009 to 2012. From 2009 to 2011, pilots flew from the North Carolina‐Virginia border (36°55′ N) to Jacksonville, Florida (30°21′ N) in February. In February 2012, pilots flew an intensive survey effort from the South Carolina‐North Carolina border (33°75′ N) to the Florida‐Georgia border (30°70′ N). The data obtained from the 2012 survey effort had not been analyzed prior to this study. Surveys consisted of east‐west transects spaced at 5 nautical mile (nm) intervals of latitude (2.5 nm in 2012). In all years except 2012, transect length was the longer of the two following distances: 14.8 km or the distance to the 16‐m depth boundary starting from the coastline and heading east as Black Scoters are unlikely to use habitats with >16‐m depth. In 2012, all transects were 18–21 nm long. Two‐person crews (pilot‐observer and observer) conducted surveys using USFWS fixed‐winged aircraft flown at ca. 70 m above sea level at 204 km/hr. The pilot‐observer and observer counted all sea ducks within 250 m of their side of the aircraft. More details about survey methodology and associated aerial survey data from 2009–2011 are described in Silverman et al. (2013a), Silverman et al. (2013b). We limited our inference to only those species identified as Black Scoters during the aerial survey.

In order to describe and quantify the habitat use of the area covered in the aerial survey, we divided transects flown by USFWS during aerial surveys into grid cells that were 1,000 m long and 550 m wide (275 m on each side of the transect) using the packages Dspat (Johnson et al., 2014), GISTools (Brunsdon & Chen, 2014), and spatstat (Baddeley et al., 2017) in R (v. 3.4.0; R Development Core Team, 2017). We used a width of 550 m to encompass the area surveyed plus an additional 25 m to each side of the surveyed area to account for possible global positioning system (GPS) error. For 2009–2012, we summed the number of Black Scoters observed in each grid cell in each year as the response variable, ranging from zero to the maximum count value observed (9,080).

### 2.2 Habitat variables

We included eight independent predictor variables in the model of Black Scoter distribution, five for modeling effects of oceanographic variables, and 3 for modeling effects of weather variables (oceanographic: bathymetry [m], ocean floor slope [degrees], distance to shore [Euclidean, km], and latitude; weather: North Atlantic Oscillation [NAO], average wind speed [m/s], and average time between waves [sec]). We also examined quadratic terms for bathymetry, distance to shore, average wind speed, and average time between waves to determine if Black Scoter presence or abundance had a nonlinear relationship with those variables. We additionally evaluated the interactive effect of NAO and distance to shore and NAO and bathymetry as scoter species may have higher abundances near shore during cold, snowy winters (negative phase NAO; Zipkin et al., 2010) and shifts in the Gulf Stream due to changes in NAO phases (Sun et al., 2020) may result in different habitat use. We did not include covariates related to weather that had previously been shown to not correlate with Black Scoter abundance (e.g., sea surface temperature, Zipkin et al., 2010). All variables were standardized to a mean value of zero and a standard deviation of 1 and collinearity between each pair of variables was less than 0.6. To calculate the value for each environmental covariate of interest, we either used the center of the grid cell (for distance to shore) or calculated the mean of a given variable for each grid cell using the raster package (Hijmans et al., 2017).

#### 2.2.1 Oceanographic covariates

We obtained bathymetric data from the National Oceanic and Atmospheric Administration's (NOAA) National Geophysical Data, ETOPO1 Global Relief Model (Amante & Eakins, 2009). We calculated ocean floor slope (degrees) by using the bathymetry data (Amante & Eakins, 2009) and calculating the difference of the values between neighboring cells (Table S1). We obtained a shoreline shapefile from NOAA's National Centers for Environmental Information Global Self‐consistent, Hierarchical, High‐resolution Geography Database (GSSH), version 2.3.6, using the intermediate resolution (i) and the boundary between land and ocean (L1; Wessel & Smith, 1996). We obtained monthly values for NAO from the Climatic Research Unit, University of East Anglia, Norwich, UK (https://crudata.uea.ac.uk/cru/data/nao/).

#### 2.2.2 Weather covariates

We obtained daily values of wind speed and time between waves for corresponding dates of aerial surveys for all grid cells from NOAA's National Data Buoy Center. We acquired wind speed data from 20 buoys located along the southeastern U.S. coast from the Virginia coast (37°60′ N) to the Florida‐Georgia border (30°70′ N). We acquired time between waves data from nine buoys located along the southeastern U.S. coast from the Chesapeake Bay (36°91′ N) to the Georgia border (31°40′ N). For each day of the aerial survey, we averaged the daily wind speed and time between waves and interpolated the average daily wind speed and time between waves across our study area by using inverse distance interpolation over the latitude range of 28° to 39° N and over the longitude range of −82° to −72° W with the gstat package (Pebesma & Graeler, 2017). Then, we averaged survey dates to calculate the average wind speed and average time between waves for each grid cell across the survey period.

### 2.3 Model fitting

We used Bayesian hierarchical models to evaluate the relationship between abundance of Black Scoters along the southern Atlantic Coast of the United States. We initially compared a zero‐inflated negative binomial, a zero‐inflated Poisson, and a negative binomial model; however, the negative binomial model was the only model to converge and have adequate model fit based on Bayesian *p*‐values. Our count data, *y_i,t_*, were modeled for each site *i* in year *t* with a negative binomial model conditional on site presenc

$$y_{i,t}∼\text{NegBinom}\left(p_{i,t},r\right)$$

where *p_i,t_* is specified as $p_{i,t}=\frac{r}{r+\mu_{i,t}}$ and *r* is the overdispersion parameter. Our mean conditional abundance, *μ_i_*_,_ *_t_*, was then modeled with a log‐linear model

$$\begin{array}{c} \text{log}\left(\mu_{i,t}\right)=\beta_{0}+\beta_{1}*{\text{NAO}}_{t}+\beta_{2}*{\text{bathymetry}}_{i}+\beta_{3}*{\text{bathymetry}}_{i}^{2}+\beta_{4}*{\text{slope}}_{i}+\beta_{5}\\ {}*{\text{distance}}_{i}+\beta_{6}*{\text{distance}}_{i}^{2}+\beta_{7}*{\text{wind}}_{i,t}+\beta_{8}*{\text{wind}}_{i,t}^{2}+\beta_{9}*{\text{wave}}_{i,t}+\beta_{10}*{\text{wave}}_{i,t}^{2}\\ +\beta_{11}*{\text{latitude}}_{i}+\beta_{12}*{\text{NAO}}_{t}*{\text{bathymetry}}_{i}+\beta_{13}*{\text{NAO}}_{t}*{\text{distance}}_{i} \end{array}$$

where **β** represents effects of covariates.

We used Bayesian *p*‐values based on the Freeman‐Tukey statistic to evaluate model fit (Conn et al., 2018). We used indicator variable selection (*γ*) to select variables for inference (Hooten & Hobbs, 2015). Because our analyses used indicator variable selection for linear, quadratic, and interactive terms, we required the model to incorporate linear terms along with higher order terms (e.g., linear terms for NAO and bathymetry were included in the interaction term for NAO × bathymetry). We incorporated these terms (**γ**_ADJ_) by multiplying all relevant *γ* values for higher order terms (e.g., value for *γ*_12_ = *γ*_1_ × *γ*_2_ × *γ*_12_). Our analyses (Supplement S1) were run in R (R Core Team, 2017) using JAGS (Plummer, 2013) via package runjags (Denwood, 2016). Because we did not have a priori information to support particular effect sizes, we used vague priors specified as *β*_0_ ~ Normal(0,0.01), **β** = **γ**_ADJ_ × *δ*, **γ** ~ Bernoulli(0.5), *δ* ~ Normal(0, *σ*), *σ* ~ InvGamma(1,1), and *r* ~ Unif(0,10).

## 3 RESULTS

**FIGURE 1:**
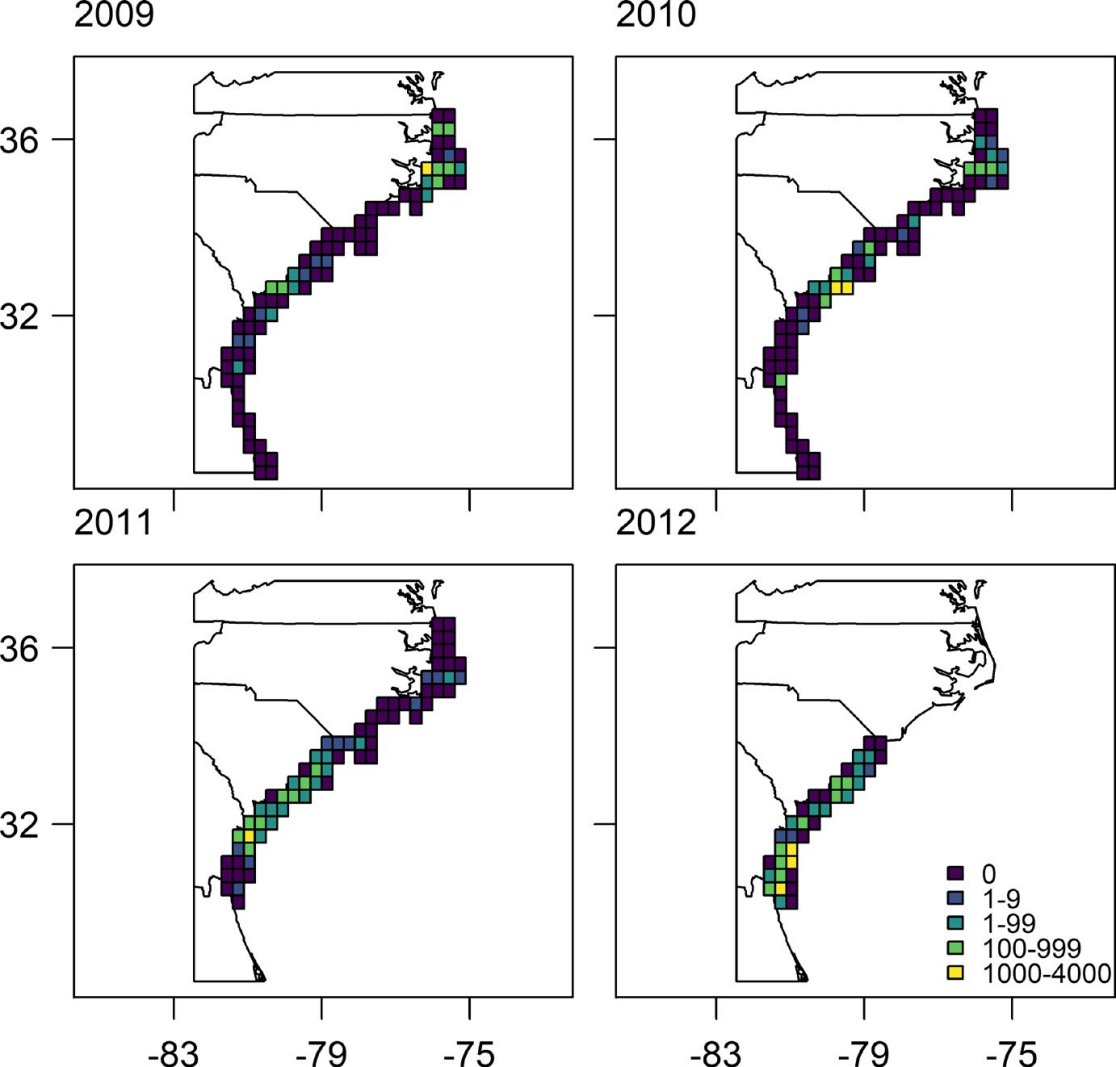
The counts of Black Scoters by year along the southeastern coast of the United States during winters from 2009 to 2012. Note that for presentation purposes, the grid cells shown are much larger (0.3 degrees) than grid cells used to model the data. Data are from U.S. Fish and Wildlife aerial surveys (Silverman et al., 2013b)

Over the four survey years, there were 16,742 grid cells sampled, of which 509 had one or more Black Scoters (Table S2). The number of Black Scoters varied annually and spatially (Figure 1). Of those cells with observed Black Scoters, seven had observations with greater than 1,000 individuals, resulting in a highly skewed distribution of counts (mean = 2.3 scoters, median = 0, 2.5% quantile = 0, 50% quantile = 0, 97.5% quantile = 2; Figure S1).

**FIGURE 2:**
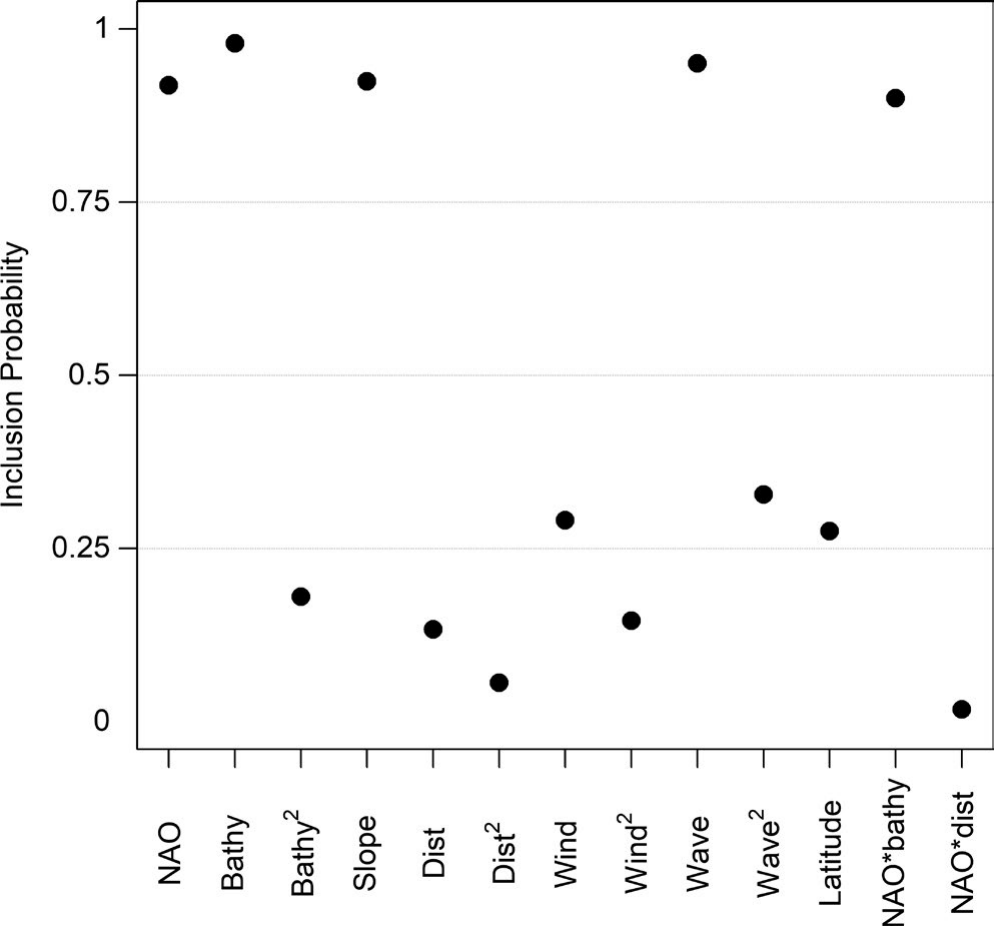
Inclusion probabilities for each of the environmental and oceanographic variables in our model for Black Scoter relative abundance during winters from 2009 to 2012. NOA = North Atlantic Oscillation, Bathy = bathymetry, Slope = ocean floor slope, Dist = distance to shore, Wind = average wind speed, Wave = average time between waves. Quadratic relationships are indicated with “^2^” and interactions with “*”

**FIGURE 3:**
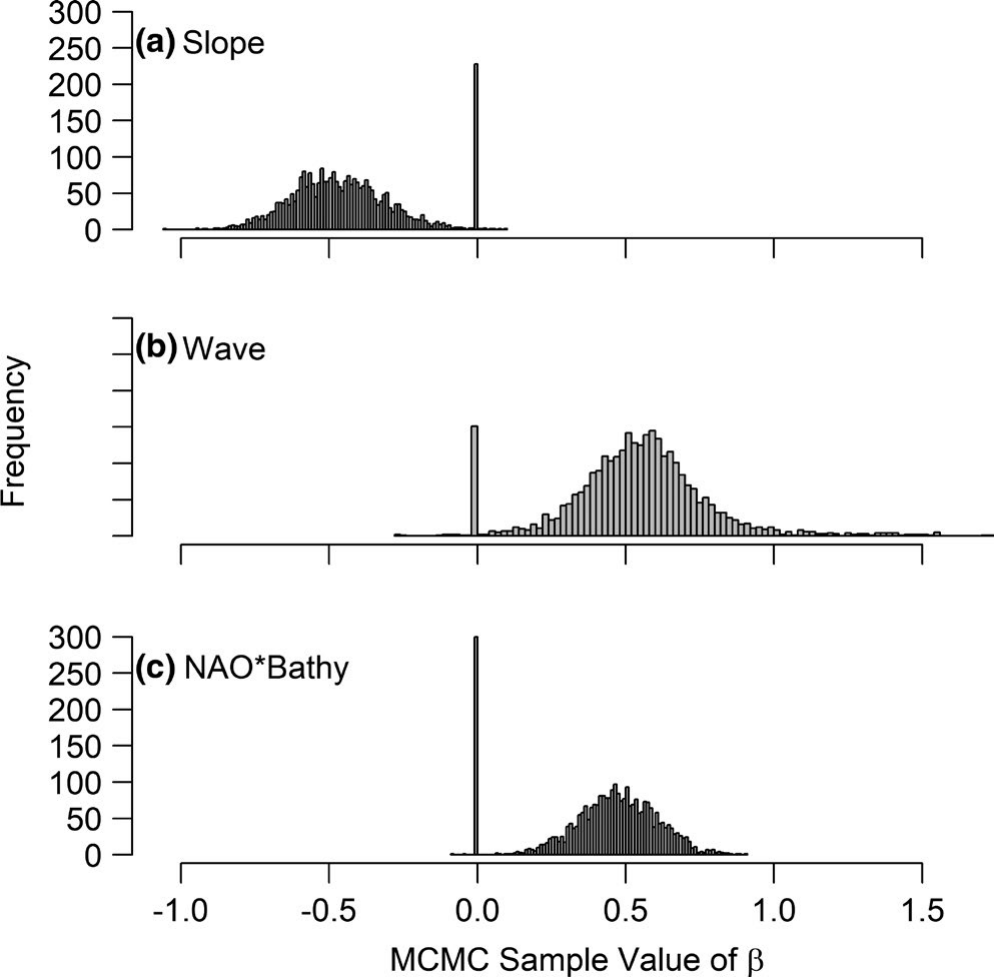
Posterior distributions of beta coefficients for models with indicator variable values greater than 0.8. Distributions are a mixture of a Gaussian distribution for those MCMC samples that include the covariate and a spike at zero for those that do not. Bathy = bathymetry, Slope = ocean floor slope, Wave = average time between waves, and NAO = North American Oscillation

The negative binomial model did not indicate any issues with model fit (Bayesian *p*‐value = 0.36), but both the zero‐inflated Poisson and zero‐inflated negative binomial models had *p*‐values < 0.1, indicating a lack of fit. The overdispersion parameter for the negative binomial model was relatively small (*r* = 0.0052, 95% Credible Intervals [CIs] = 0.0047–0.0057). The model indicated that the covariates for bathymetry (*P*[*β* > 0] = 0.92), slope (*P*[*β* > 0] = 0.001), time between waves (*P*[*β* > 0] = 0.77), and an interaction between NAO and bathymetry (*P*[*β* > 0] = 0.96) were likely greater or less than 0 and should be included in the model (Figures 2 and 3).

**FIGURE 4:**
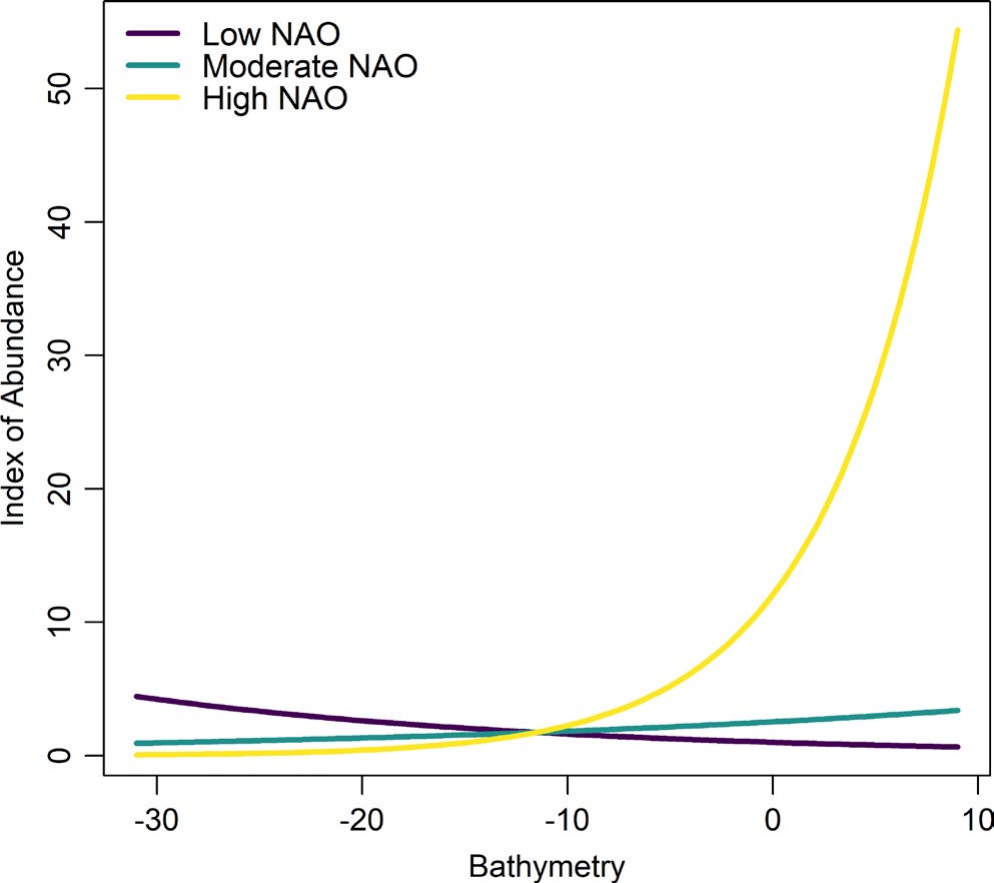
Predicted Black Scoter relative abundance in response to the interaction between bathymetry (m) and the North Atlantic Oscillation (NAO) along the southeastern coast of the United States during the winter of 2009. Three values of NAO are shown: low NAO = −3.9, moderate NAO = −1.4, and high NAO = 2.8

**FIGURE 5:**
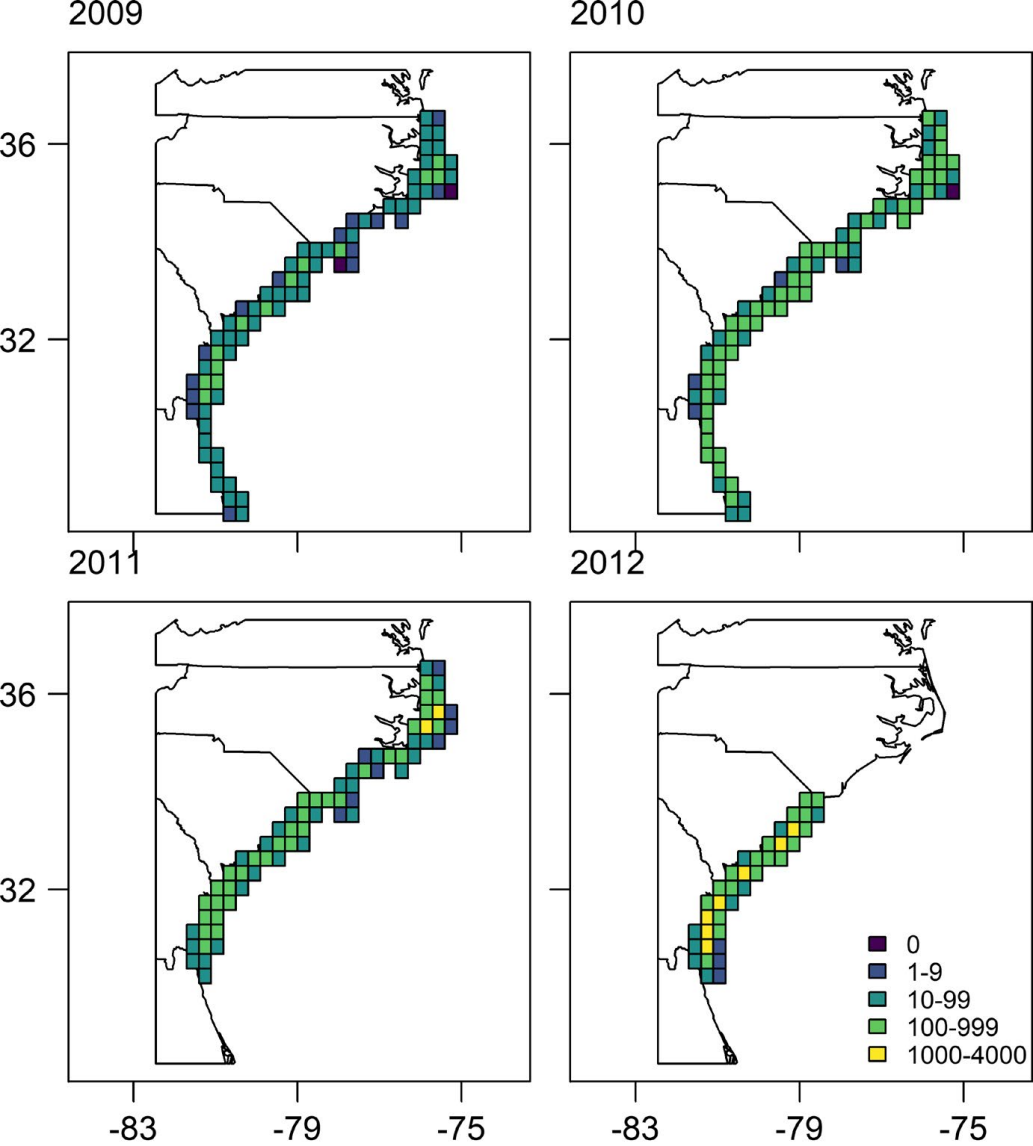
Predicted relative abundance (number of scoters) from a model for wintering Black Scoters along the southeastern coast of the United States from 2009 to 2012. Note that for presentation purposes, the grid cells shown are much larger (0.3 degrees) than grid cells used to model the data

Our model indicated that Black Scoter abundance was greater in areas with greater time between waves and flatter slopes. Additionally, the response of Black Scoter to bathymetry was modulated by changes in NAO. In years with lower NAO (cooler, wetter years), abundance was higher in deeper waters, whereas it was higher in shallower waters in years with high NAO (Figure 4). Predictions from the model indicated that abundance was highest along the coast of North Carolina in 2011 and the coast of Georgia and Florida in 2012, and lower overall in 2009 and 2010 (Figure 5).

## 4 DISCUSSION

Few studies have examined sea duck habitat use along the southeastern Atlantic Coast of North America (Bowman et al., 2015; Jodice et al., 2013; Kaplan, 2011). Our study extends early descriptions (Silverman et al., 2013b) by developing models that explore the relative importance of habitat on Black Scoter distribution from North Carolina to the northern coast of Florida. Our results indicate that a combination of fine‐scale habitat (the interaction of NAO and bathymetry and ocean floor slope) and weather conditions (time between waves) is associated with Black Scoter wintering abundance in the Southeast United States.

Along the southeastern coast of the United States, Black Scoters abundance was greatest in deep waters (depth of 30 m) during years of negative phase NAO values (cooler, drier years). During years with positive phase NAO, Black Scoters were more abundant in shallower waters. Water depth in the South Atlantic Bight increases slowly as distance to shore increases, perhaps indicating that Black Scoters prefer these shallower waters in positive phases of NAO and cooler waters further north during negative NAO phases. The rapid increase in water depth at higher latitudes limits the area accessible to Black Scoters for diving and foraging in contrast to the flat topography at lower latitudes that allows scoters to use areas further away from shore, as shown by our negative relationship between slope and relative abundance (Silverman et al., 2013; Zipkin et al., 2010). While the biological mechanism for shifts in Black Scoter use of different ocean depths in response to changes in NAO is unclear, broad‐scale changes in environmental conditions driven by the Gulf Stream in response to NAO phase (Sun et al., 2020) may be responsible for shifts in different habitat use by Black Scoters.

In addition to bathymetry, NAO, and slope, time between waves also affected Black Scoter wintering distribution. Greater wind and wave speeds may have negative effects on energetic costs for sea ducks (Žydelis & Richman, 2015), causing them to seek out calmer waters. Additionally, increased time between waves may increase observability of Black Scoters, resulting in greater counts in these conditions.

We did not find strong relationships between Black Scoter habitat use and wind speed, distance to shore, or latitude, though these have been shown to affect Black Scoters in more northern studies using satellite telemetry (Smith et al., 2019). These differences could be due to the nature of the aerial survey and the “snapshot” in time that it captures. Our aerial survey also was unable to account for detection probability which might affect perceived habitat use if the probability of detecting a Black Scoter correlated with our habitat covariates. Additionally, the lack of relationships with these covariates could be due to differences in habitat use at different latitudes (Plumpton et al., 2020).

Overall, we examined a suite of oceanographic and weather variables that aimed to explain the annual variation in wintering distribution of Black Scoters. We were able to identify several key variables that influenced Black Scoter wintering distribution along the southeastern coast of the United States, primarily related to shifts in habitat use concurrent with NAO phase and fine‐scale responses in abundance to average time between waves. These results lead to several conclusions: (a) In addition to NAO, Black Scoters may be responding to ephemeral habitat variables that we are not able to measure, such as prey abundance and distribution, (b) despite the large survey effort, the dispersed and variable distribution of Black Scoters requires additional survey data in order to develop predictive models and identify key variables (Kinlan et al., 2012), and (c) managers may need to consider large sections of the coast as important areas to wintering Black Scoters given that habitat use can shift based on NAO phase. One further area of investigation would be evaluating diet and prey resources of Black Scoters in the southeast. There have been several studies examining diet of Black Scoters along the Atlantic Coast of the United States (Stott & Olson, 1973; Goudie & Ankey 1988; Loring et al., 2013; Perry et al., 2007), but none along the southeastern coast. Contrasting diet of Black Scoters wintering in the southeast relative to those wintering further north could help identify important food resources and provide greater insight into drivers of the wintering distribution of Black Scoters. Additional survey work paired with data collection targeting possible Black Scoter prey and location‐specific environmental conditions could further reduce uncertainty associated with Black Scoter wintering habitat use.

Our study builds on previous work that provided a description of Black Scoter distribution and variability (Silverman et al., 2013a). By using a modeling approach to determine an important set of variables to help describe and predict Black Scoter wintering distribution, we are able to provide managers with information to help predict potential wintering habitats for Black Scoters in the southern portion of the Atlantic Coast of the United States. The identification of key habitat associations, such as the relationship of bathymetry and NAO, provides valuable insight into Black Scoter annual wintering distribution and habitat use. Due to large unexplained variation in annual abundance, additional data are needed on Black Scoter distribution and abundance in relation to prey preference, the distribution of prey species, and the substrate types where the prey species are found. Additional data would refine our understanding of critical habitats and habitat use, help identify areas of high‐quality wintering habitat and resources, and benefit efforts to protect key areas. Understanding wildlife population distribution and dynamics as they relate to habitat use allows for more effective conservation planning, may minimize human conflicts, and could benefit survey planning for future monitoring programs (Newbold & Eadie, 2004; Rushing et al., 2017).

## CONFLICT OF INTEREST

We declare no competing interests.

## AUTHOR CONTRIBUTIONS

**Hannah M. Plumpton:** Conceptualization (equal); formal analysis (lead); methodology (equal); writing‐original draft (lead); writing‐review & editing (lead). **Emily D. Silverman:** Conceptualization (supporting); data curation (lead); methodology (supporting); writing‐review & editing (supporting). **Beth E. Ross:** Conceptualization (equal); methodology (equal); supervision (lead); writing‐original draft (supporting); writing‐review & editing (supporting).

## Supporting information

Code S1

Appendix S1

## Data Availability

Data used for the analysis are uploaded in a Dryad repository (https://doi.org/10.5061/dryad.31zcrjdkj).
