# Supplementary material for: Black Scoter habitat use along the southeastern coast of the United States
Source: Ecol Evol. 2021 Jul 27;11(16):10813–20. doi: 10.1002/ece3.7746 (PMC8366858; doi:10.1002/ece3.7746)
Supplement: Supplementary file 2 — Appendix S1 [file ECE3-11-10813-s002.docx]

APPENDIX

Table A1: The eight covariates examined in relation to Black Scoter abundance along the southeastern coast of the United States during the winters from 2009 to 2012, the covariates’ spatial scale or resolution and the source of variable.

| Variable | Spatial or Temporal? | Scale/Resolution | Mean | Standard Deviation | Range | Source |  |
| --- | --- | --- | --- | --- | --- | --- | --- |
| Bathymetry (water depth) | Spatial | WGS 84  1 arc-minute (cell size) | -10.99 m | 5.50 | -31.00 – 9.00 | NOAA National Centers for Environmental Information (Amante and Eakins 2009) |  |
| Average Wind Speed | Spatial and temporal | WGS 84  1 arc-minute | 5.00 m/s | 0.62 | -4.16 – 5.91 | NOAA National Data Buoy Center |  |
| Average Time between Waves | Spatial and temporal | WGS 84  1 arc-minute | 4.99 sec | 0.14 | 4.80 – 5.14 | NOAA National Data Buoy Center |  |
| North Atlantic Oscillation | Temporal | Monthly Value, Feb 2009 to Feb 2012 | -0.33 | 2.59 | -3.92 – 2.79 | Climatic Research Unit, University of East Anglia, Norwich, UK |  |
| Distance to Shore | Spatial | Euclidean distance between Black Scoter location and edge of the Atlantic coastline | 17351 m | 13323 | 1369 – 69516 | NOAA National Centers for Environmental Information GSHHG data (shoreline data) |  |
| Ocean Bottom Slope | Spatial | Calculated from bathymetry layer, difference of the values between neighboring cells | 0.05 degrees | 0.05 | 0.00 – 0.41 | NOAA National Centers for Environmental Information ETOPO1 data |  |

Table A2: The mean, minimum, and maximum count of Black Scoters observed and the number of grid cells where Black Scoter abundance was ≥ 1 during each survey year in a given cell among all surveyed cells, during the winters from 2009 to 2012 along the southeastern coast of the United States. Data are from U.S. Fish and Wildlife aerial surveys (Silverman et al. 2013).

| Survey Year | Mean | Minimum | Maximum | No. of Occupied Cells |
| --- | --- | --- | --- | --- |
| 2009 | 39.75 | 1 | 475 | 80 |
| 2010 | 100.40 | 1 | 9,080 | 157 |
| 2011 | 66.63 | 1 | 2,910 | 164 |
| 2012 | 93.94 | 1 | 2,900 | 108 |

Figure A1: Histogram (all counts [A], all counts > 0 [B], and log scale of counts > 0 [C]) of Black Scoters observed during aerial surveys from 2009 to 2012 along the southeastern coast of the United States.


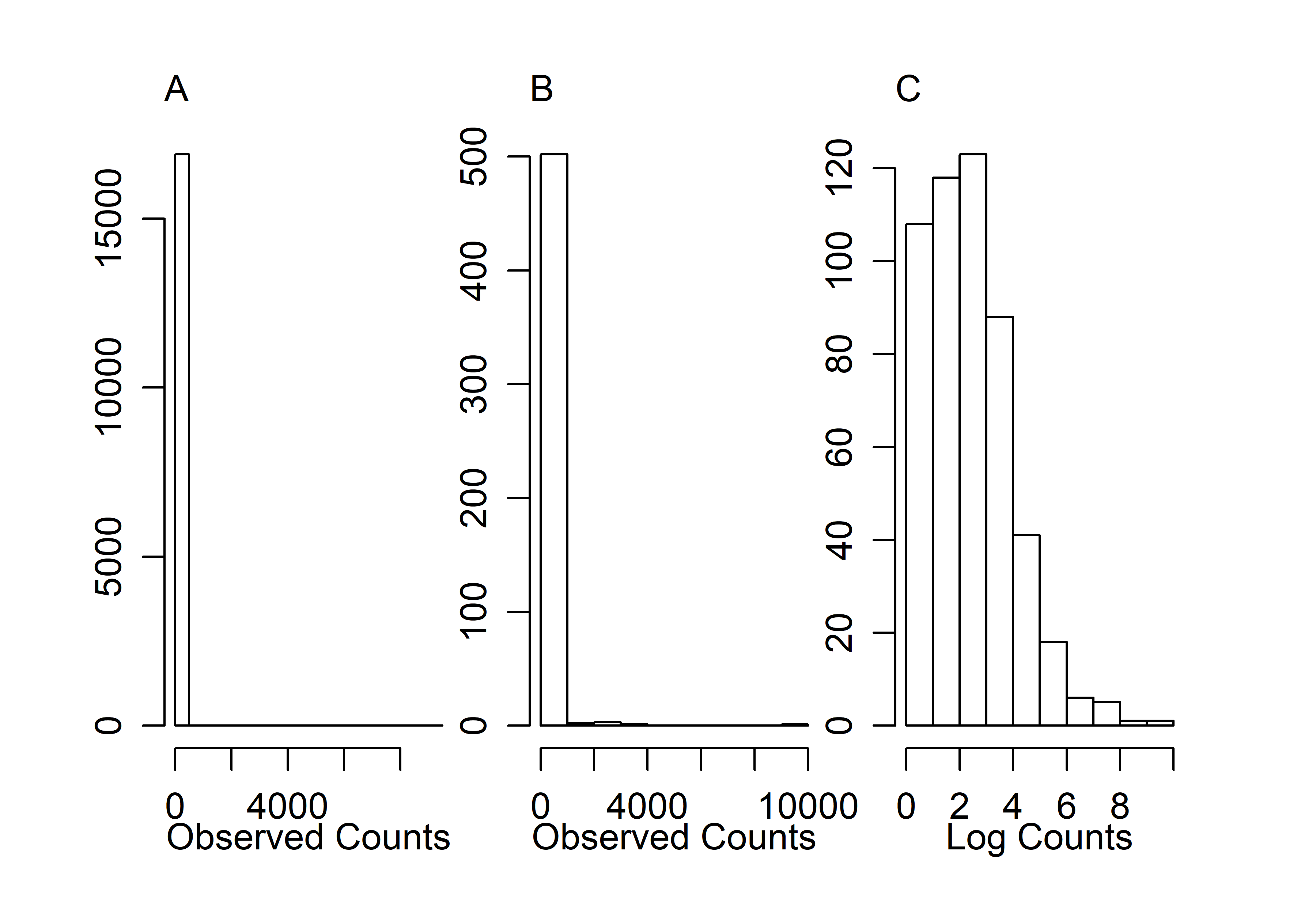
Literature Cited

Amante, C. and B.W. Eakins. 2009. ETOPO1 1 arc-minute global relief model: procedures, data sources and analysis, National Geophysical Data Center, NESDIS, NOAA, U.S. Dept. Commerce, Boulder, CO, USA.

Jones, P. D., Jónsson, T., and D. Wheeler. 1997. Extension to the North Atlantic Oscaillation using early instrumental pressure observations from Gibraltar and South-West Iceland. International Journal of Climatology 17:1433–1450. https://crudata.uea.ac.uk/cru/data/nao/

Silverman, E. D., D. T. Saalfeld, J. B. Leirness, and M. D. Koneff. 2013b. Data from: Wintering sea duck distribution along the Atlantic coast of the United States. Journal of Fish and Wildlife Management 4:178–198. Archived in Dryad Digital Repository: http://dx.doi.org/10.5061/dryad.m9t12.

US DOC/NOAA/NWS/NDBC. National Data Buoy Center. 1971. Meteorological and oceanographic data collected from the National Data Buoy Center Coastal-Marine Automated Network (C-MAN) and moored (weather) buoys. NOAA National Centers for Environmental Information. https://www.ndbc.noaa.gov/

Wessel, P., and W. H. F. Smith. 1996. A global, self-consistent, hierarchical, high-resolution shoreline database. Journal of Geophysical Research: Solid Earth 101:8741–8743. https://doi.org/10.1029/96JB00104
